# Life Satisfaction of Nurses during the COVID-19 Pandemic in Poland

**DOI:** 10.3390/ijerph192416789

**Published:** 2022-12-14

**Authors:** Anna Stefanowicz-Bielska, Magdalena Słomion, Małgorzata Rąpała

**Affiliations:** 1 Division of Internal and Pediatric Nursing, Institute of Nursing and Midwifery, Faculty of Health Sciences with the Institute of Maritime and Tropical Medicine, Medical University of Gdansk, 80-211 Gdansk, Poland; 2 Department of Pediatric Surgery, Marciniak Hospital, 50-041 Wroclaw, Poland

**Keywords:** nurses, life, satisfaction, SARS-CoV-2, infections, COVID-19, occupational stress

## Abstract

Background: Health care practitioners are at highest risk of COVID-19 disease. They experience an enormous overload of work and time pressures. The objective of the study was to assess nurses’ life satisfaction. Method: The study included professionally active nurses. The research method was an author’s questionnaire and a standardized questionnaire, the Satisfaction with Life Scale (SWLS). Results: The study group included 361 working nurses. The mean raw score and the sten score of the nurses’ responses to the statements on the SWLS questionnaire were 21.0 (SD ± 5.6, range = 5–35) and 5.73 (SD ± 1.94, range = 1–10), respectively. It was shown that lower life satisfaction was experienced by nurses aged 51 to 60 (raw score: p = 0.003, sten score: p = 0.005), as well as nurses with secondary and undergraduate nursing education (raw score: p = 0.061, sten score: p = 0.043). Nurses who had a higher self-evaluation of the level of knowledge about SARS-CoV-2 infection experienced greater life satisfaction (raw score: p = 0.008, sten score: p = 0.022). Conclusions: The majority of Polish nurses surveyed during the COVID-19 pandemic had a low or medium level of life satisfaction. The low response rate to the survey was most likely due to work overloads during the pandemic. Working in a public service profession, a nurse is exposed to stressful conditions related to protecting human health. Constant difficult and stressful situations and total fatigue experienced by nursing professionals can be the cause of a lack of motivation, occupational burnout, listlessness and mental and physical disease. Further research is necessary to assess the factors positively influencing the level of life satisfaction.

## 1. Introduction

In December, 2019, a virus that commonly causes acute pneumonia was detected in China [1]. The SARS-CoV-2 virus has affected the health of many people [2,3].

Due to the severity of disease complications and its high mortality, it was necessary to hospitalize patients and to provide them with specialty care [4,5].

The COVID-19 pandemic was, and continues to be, extremely difficult for health care practitioners, due to the disease’s high contagiousness and the severity and complications of the infection [6].

Health care practitioners are the group most affected in the fight against the SARS-CoV-2 virus [7].

Nurses, midwives and doctors represent the largest group of medical staff engaged in the fight against the COVID-19 pandemic. They experience an enormous overload of work and time pressures. They often experience rudeness in the workplace, a lack of autonomy and social support, isolation from their families, discrimination, fake news and conspiracy theories [8,9,10,11,12].

Nursing is a profession of great social importance. The changing conditions of daily work, mental and physical stress, conflicts in the therapeutic team, and shift work may result in a lower level of life satisfaction in this professional group. Satisfaction with life is an element of quality of life. Many factors can affect satisfaction with life for nurses, including insufficient financial and psychological compensation, personality, life events and current mood [13].

Nurses caring for COVID-19 patients encountered stressors related to working conditions, difficulties with interpersonal communication, lack of support from coworkers, isolation, social stigma, fear of transmitting the disease to their relatives and high level of stress and fear in the workplace [14]. Nurses had to often make difficult decisions that could cause ethical dilemmas [15].

Sleep disorders can also be a consequence of the stresses encountered [16]. During stress, the quality of sleep may deteriorate [16,17]. During the COVID-19 pandemic sleep disorders affected about 40% of professionally active nurses and midwives in Poland [18]. People who report a lack of sleep or insomnia have less control of behavior and reactions. Abnormal sleep and insomnia affect well-being and satisfaction with life [16,19].

The prevalence of sleep disturbances, moderate anxiety and low self-efficacy among Italian nurses was 71.4%, 33.2% and 50.6%, respectively in one study [20].

Common psychological effects of the pandemic included anxiety, panic, depression, anger, confusion, addictions, post-traumatic stress, ambivalence, the psychological impact of financial stress and increased risk of occupational burnout. Depression, anxiety and posttraumatic stress disorder were the most common mental disorders that were reported among health care practitioners during the pandemic [14,21,22]. Worldwide, approximately one third of nurses working during the COVID-19 epidemic were suffering from psychological symptoms [23].

Health care practitioners who care for COVID-19 patients are exposed to particular stressors and posttraumatic stress disorders in the workplace. Working with COVID-19 patients causes anxiety, anger, depression and mental crisis.

The state of emotional tension and stress caused by exhausting working conditions can lead to professional burnout [24].

The risk of infection and the difficult working conditions during the pandemic could significantly reduce life satisfaction of nurses [25].

The objective of the study was to assess the level of nurses’ life satisfaction.

## 2. Materials and Methods

### 2.1. Study Design, Setting and Participants

A study was conducted from 1 April 2021 to 30 June 2021, during the pandemic.

**Figure 1:**
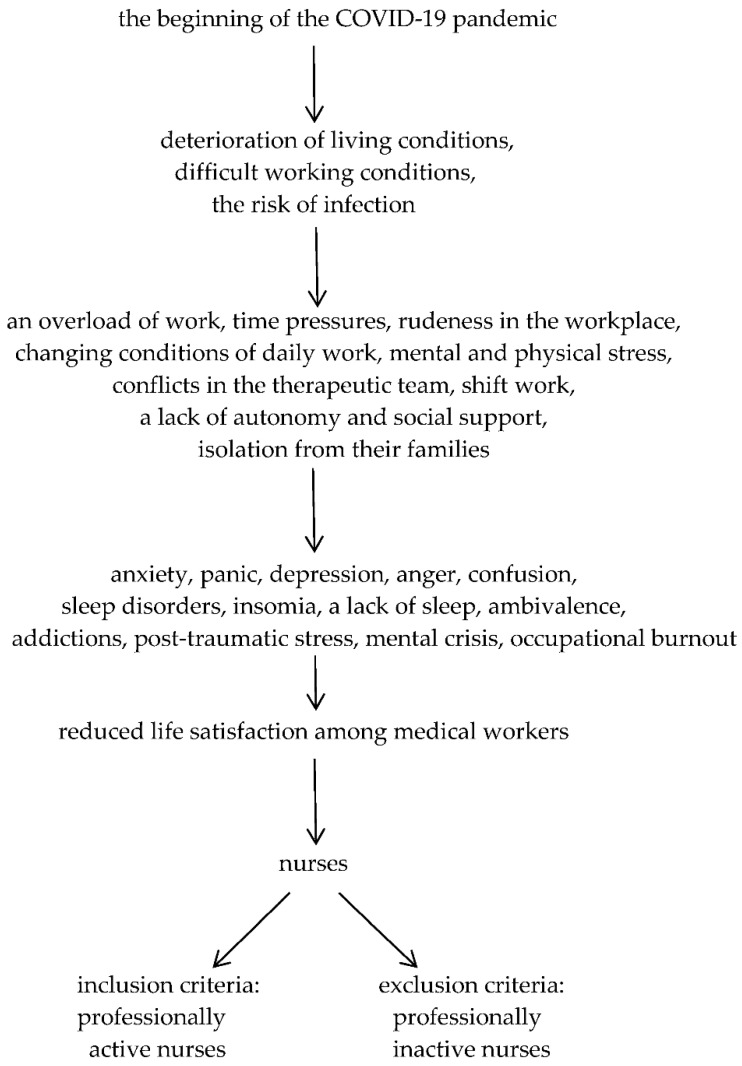
Study design.

The participants of the study were professionally active nurses (Figure 1). Twenty-one selected health care entities in Poland were invited. Only 14 health care entities confirmed their participation in the study. 

Participants of this study also participated in a study about strategies for managing stress [26].

### 2.2. Methods

We conducted a study using a survey, which included an author’s questionnaire and the standardized questionnaire, the Satisfaction with Life Scale (SWLS), created by Diener and et al. [27,28].

#### 2.2.1. An Author’s Questionnaire

The questionnaire included questions to gather sociodemographic data, and questions concerning the possibility of contact with COVID-19 disease and self-assessment of knowledge about SARS-CoV-2 virus infection.

#### 2.2.2. The Satisfaction with Life Scale (SWLS)

The SWLS contains five statements: 1. In most ways, my life is close to my ideal. 2. The conditions of my life are excellent. 3. I am satisfied with my life. 4. Thus far, I have gotten the important things I want in life. 5. If I could live my life over, I would change almost nothing [28,29,30].

Each nurse participating in the survey indicated to what extent each statement related to his or her current life. Each statement was scored from 1 to 7, where 1 meant “definitely not in agreement” and 7 meant “strongly in agreement”. The total SWLS score was calculated as the sum of all five items [29].

The result of the measurement indicated the level of satisfaction with one’s own life. The total score could range from 5 to 35 points, representing a raw result. The higher the score, the higher the sense of satisfaction with life [29].

Raw scores were transformed into standard units on the sten scale [29].

Properties characterizing the sten scale should be considered when interpreting the results. Sten scores from 1 to 4 were considered low, and those from 7 to 10 were considered high. Results of 5 and 6 were considered medium [29]. 

The electronic version of the SWLS was used with the permission of the Psychological Test Laboratory of the Polish Psychological Association.

### 2.3. Data Collection

The data were collected using an electronic survey. Links to the survey were sent to all nurses via email by heads of the participating hospitals. Participation in the survey was voluntary and anonymous.

### 2.4. Ethical Considerations

The study protocol was approved by the Independent Bioethics Committee for Scientific Research of the Medical University of Gdansk. We also obtained approval from the Directors of each hospital. Nurses were enrolled after giving their informed consent. Data confidentiality was ensured during the data collection and data analysis phases.

### 2.5. Statistical Analysis

For each parameter mean (X), median (M), standard deviation (SD, range (min, max), and lower and upper quartiles (25Q, 75Q) were calculated. Statistical significance between means for different groups was calculated by means of one-way analysis of variance (ANOVA), alternatively using the non-parametrical U Mann–Whitney test (for two groups) or Kruskal–Wallis test (for more than two groups), when the variances in groups were not homogeneous (the homogeneity of variance was determined by the Levene’s test).

Statistical significance between frequencies was calculated by the chi-square test χ^2^_df_ with corresponding degree of freedom df (df = (m − 1) * (n − 1), where m referred to number of rows, and n to number of columns.

A p value of less than 0.05 was required to reject the null hypothesis. Statistical analysis was performed using EPIINFO Ver. 7.2.3.1 and Statistica Ver. 13.3. software packages.

## 3. Results

### 3.1. Characteristics of the Nurses

Participation in the study was confirmed by 361/6560 (5.5%) nurses (F:M = 342:19). A total of 6199 nurses refused to participate in the study. Most of the nurses were women (94.7%), between the ages of 51 and 60 (33%) and lived in a city (83.7%). A total of 45.7% of the nurses had a master’s degree in nursing (Table 1).

The nurses participating in the study worked in outpatient medical units (17.5%), paediatric units (16.9%), surgical units (15.8%), internal diseases units (13.3%), surgical wards (9.1%), intensive care wards for adults (7.8%), emergency wards (5.8%), intensive care wards for children (3.6%), hospices (3.6%), psychiatric units for adults (3.1%), medical universities (1.9%), health care and curative institutions (1.4%) and psychiatric units for children (0.2%) (Table 1).

The participants were divided into groups of nurses working in clinics (17.5%), hospital wards (75.6%) hospices and health care and curative institutions (5%), and medical universities (1.9%) (Table 1). Hospital nurses were divided into nurses working in paediatric units (22.3%), internal diseases units (17.6%), surgical wards and surgical units (33%), intensive care (15%), emergency wards (7.7%) and psychiatric units for adults and children (4.4%).

As many as 61.5% of the participants had ≥21 years of employment in the profession.

The nurses estimated that their level of knowledge about COVID-19 and the principles of prevention and dealing with an infected patient was high (50.7%), medium (47.9%) or low (1.4%). As many as 74.8% of nurses had close contact with COVID-19 disease.

### 3.2. Satisfaction with Life

The results of the SWLS psychological questionnaire for the individual nurses were analysed. The nurses’ responses to the statements in the SWLS are presented in Table 2.

The mean raw score and sten score of the nurses’ answers to the statements in the SWLS psychological questionnaire were 19.2 (standard deviation ± 5.6, 5–35) and 5.06 (standard deviation ± 1.94, 1–10), respectively.

The number of nurses who obtained the various levels of life satisfaction, according to the results of the SWLS, is presented in Table 3.

As many as 23% of nurses felt low satisfaction with life, 44.1% had a medium level of life satisfaction, and 32.9% had a high level of life satisfaction.

### 3.3. Influence of Various Factors on Satisfaction with Life

The level of life satisfaction did not depend on sex (raw score: *p* = 0.981, sten score: *p* = 0.932), place of residence (raw score: *p* = 0.618, sten score: *p* = 0.542), place of work (outpatient clinic vs. hospital vs. hospice and health care and curative institution vs. medical university; raw score: *p* = 0.137, sten score: *p* = 0.096) or years of employment in the profession (raw score: *p* = 0.669, sten score: *p* = 0.513). In the case of nurses employed in hospital departments, there was no impact of the type of ward on the level of life satisfaction (raw score: *p* = 0.216, sten score: *p* = 0.221) (Table 4).

**Figure 2:**
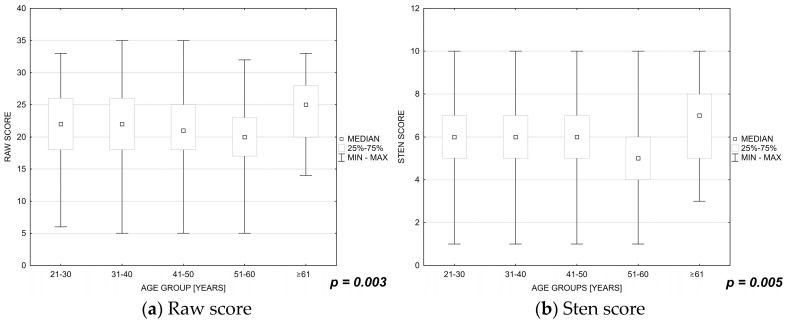
(**a**,**b**) Influence of the nurses’ ages on their satisfaction with life.

**Figure 3:**
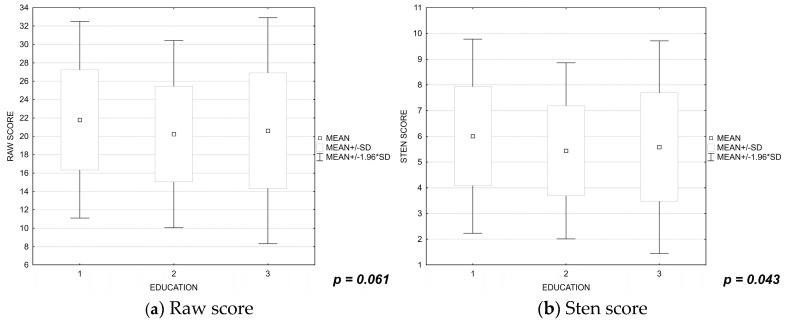
(**a**,**b**) Influence of the nurses’ levels of education on their satisfaction with life. 1—Master of nursing, 2—Bachelor of nursing, 3—Secondary education in nursing.

It was shown that lower satisfaction with life was experienced by nurses aged 51 to 60 years (raw score: *p* = 0.003, sten score: *p* = 0.005; Figure 2a,b) and nurses with secondary and undergraduate nursing education (raw score: *p* = 0.061, sten score: *p* = 0.043; Figure 3 a,b).

**Figure 4:**
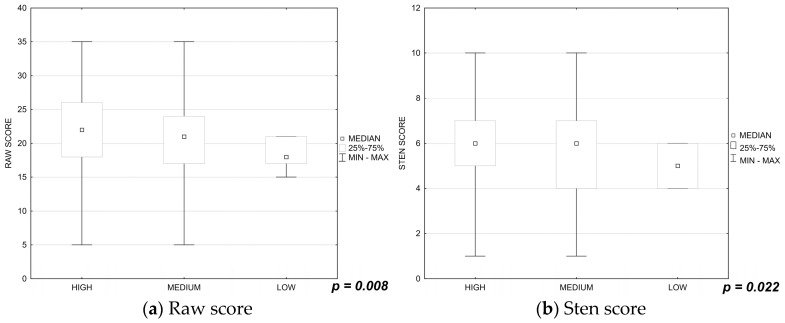
(**a**,**b**) Influence of the nurses’ self-assessed knowledge of SARS-CoV-2 infection on their satisfaction with life.

The nurses whose self-assessed knowledge of SARS-CoV-2 infection was higher felt greater satisfaction with life (raw score: *p* = 0.008, sten score: *p* = 0.022; Figure 4a,b).

Contact with COVID-19 disease did not affect the nurses’ level of life satisfaction (raw score: yes—20.9 ± 5.8, no—21.5 ± 5.2, *p* = 0.430, sten score: yes—5.83 ± 1.83, no—5.85 ± 1.79 *p* = 0.524).

## 4. Discussion

The worldwide outbreak of the COVID-19 pandemic induced, and revealed, many emotions among medical workers. Health care practitioners were forced to work in particularly difficult conditions [31]. Caring for patients with SARS-CoV-2 affected the mental health of health care professionals. Many health care professionals experienced mental disorders and trauma, and needed psychological support [25]. It was, therefore, important to assess the life satisfaction of nurses during the pandemic.

The objective of the work was to assess the level of nurses’ life satisfaction.

A questionnaire was administered to 6560 Polish nurses, but only 361 (5.5%) professionally active nurses responded. Such a low level of responses was puzzling. It was most likely caused by fatigue and/or stress related to the difficult working conditions during the pandemic.

Many researchers assessed nurses’ life satisfaction before the COVID-19 pandemic.

Ghazwin et al. [32] assessed life satisfaction among Iranian nurses employed in three academic hospitals. Ninety-four nurses participated in the study. Forty-five percent of the participants were not satisfied with their lives. Severe depression was associated with a low level of life satisfaction. The study showed that poor satisfaction with life associated with financial status and the working environment. Depression, anxiety and stress were the main determinants of low satisfaction with life [32].

In that study, the SWLS scale showed extremely low life satisfaction in 14.9% of respondents [32].

In our study, 23% of respondents showed low satisfaction with life.

In the Iranian study, medium satisfaction with life was reported by 16% of respondents, and the most commonly observed score range of the assessment scale was 10–14, representing 29.8% of respondents (vs. 23% in our study). Only 11.7% of participants reported a high level of satisfaction with life, while in our study, 32.9% had a high level of satisfaction. Higher life satisfaction was associated with financial satisfaction and with satisfaction with working conditions. Lower life satisfaction was associated with severe depression, anxiety and stress [32].

Sanso et al. [33] assessed life satisfaction in 210 Spanish nurses from the Healthcare Public System of the Balearic Islands. The mean life satisfaction score was 3.3 ± 0.89. The authors suggested that the life satisfaction level before the COVID-19 pandemic was medium to high [33].

A Polish study conducted by Zborowska et al. [34] included 625 nurses whose life satisfaction was assessed using the SWLS and whose professional satisfaction was assessed with the Satisfaction with Job Scale. A total of 218 (37.8%) nurses had a low level of satisfaction with life, 147 (25.5%) had a medium level, and 212 (36.7%) had a high level [34].

In our study, conducted in the era of the COVID-19 pandemic, 23% of respondents had low life satisfaction, 44% had medium life satisfaction, and 32.9% had high life satisfaction. It was puzzling why, in a study of the same population before the COVID-19 pandemic, life satisfaction was worse than life satisfaction during the COVID-19 pandemic.

A study conducted by Uchmanowicz et al. [35] in a group of Polish active nurses (*n* = 350) and midwives (*n* = 57) undergoing specialisation training in Wrocław showed that 160 (46%) participants had high life satisfaction, 128 (37%) had medium life satisfaction, and 62 (18%) had low life satisfaction [35].

An interesting issue is the assessment of nurses’ level of satisfaction with life during the COVID-19 pandemic and the answer to the question of how the COVID-19 pandemic influenced nurses’ satisfaction with life.

Karabağ Aydin et al. [36] assessed life satisfaction in Turkish nurses during the COVID-19 pandemic. A total of 411 nurses working in public and private health care entities participated in the study. More than half of the nurses stated that in the clinic where they worked, they had contact with a COVID-19 patient (56%) and were concerned about caring for COVID-19 patients (54.5%). In this study, nurses had low life satisfaction during the COVID-19 pandemic. The mean total SWLS score was 12.91 (standard deviation = 4.11; range = 5–25), and the item score was 2.58 (standard deviation = 0.82; range = 1–5). There were very weak negative correlations between the level of life satisfaction and age, male sex, frequency of thinking about death, frequency of encountering death in the unit where the nurse worked, and death anxiety. In contrast, the level of life satisfaction tended to be higher among nurses who were not afraid of caring for COVID-19 patients [36].

In our study, the degree of satisfaction with life did not depend on sex, place of residence, place of work, or job seniority. We found that nurses aged 51 to 60 and those with secondary and undergraduate nursing education experienced lower life satisfaction. Meanwhile, nurses who had higher self-esteem regarding their level of knowledge about SARS-CoV-2 infection experienced greater satisfaction with life.

An Iranian study by Zakeri et al. [25] involved 185 nurses directly caring for COVID-19 patients. The Iranian researchers found that 50.3% of nurses experienced mental disorders, 68.1% experienced social impairment, 49.2% showed somatic symptoms, 49.7% had anxiety and insomnia, and 18.4% had severe depression. The mean score of satisfaction with life was 23.60 ± 6.14 (range = 8–35). Of 185 participants, 84 (45.4%) were satisfied/very satisfied with their lives. The bivariate analysis showed a significant association between psychosocial disorders, sex, work experience, being at risk of contracting the coronavirus infection, and satisfaction with life. The authors showed that being at risk of contracting the coronavirus infection, having low satisfaction with life and lacking resilience were significantly associated with psychological disorders. The risk of psychological disorders was 2.56 times higher in nurses who were not at risk of coronavirus infection, than in those who were at risk of coronavirus infection (95% confidence interval for odds ratio: 1.05–624, *p* = 0.04). The risk of psychological disorders was 2.42 times higher in nurses who were poorly/not satisfied with their lives than in those who were satisfied/very satisfied with their lives (95% confidence interval for odds ratio: 1.15–5.07, *p* = 0.02) [25].

Zhang et al. [37] examined the predictors of job satisfaction, life satisfaction, and turnover intention of health care workers during the COVID-19 pandemic. A total of 240 medical workers from Bolivia in South America participated in the study. The results revealed that the number of office days predicted job satisfaction, life satisfaction, and turnover intention, but the relationships varied by age. Health care workers’ office days negatively predicted job satisfaction for the younger workers but positively predicted job satisfaction for the older workers. This study demonstrated that health care workers’ number of office days was relevant to their job satisfaction, life satisfaction, and turnover intention [37].

An interesting study on the assessment of life satisfaction in individual professional groups in health care during the COVID-19 pandemic is the work of Teke et al. A survey was conducted on 560 health care workers, including 367 nurses, 46 doctors, 61 midwives and others. The authors did not show any differences between the professional groups. The mean raw score for life satisfaction was 20.64 ± 6.18 for nurses, 22.54 ± 5.58 for doctors, 21.08 ± 4.16 for midwives, 22.2 ± 7.73 for physiotherapists, and 24.75 ± 2.76 for others. These results were similar to our results, where the mean raw score was 21.0 ± 5.6. Based on this study, it could be concluded that, during the COVID-19 pandemic, the type of medical profession had no impact on life satisfaction [38].

Turkish researchers showed that the level of education affected the degree of life satisfaction. The highest degree of life satisfaction was experienced by people with a doctorate degree (23.36 ± 6.03), and the lowest was experienced by those with a bachelor’s degree (20.26 ± 6.44, *p* = 0.002) [38].

In summary, it is difficult to compare our results with the results of other researchers, as the studied populations differ in many sociodemographic and social factors.

We were surprised by the low rate of responses to the questionnaire: 6560 questionnaires were sent, and 361 (5.5%) nurses responded. In the study by Ghazwin et al. [32], 94 (94%) nurses answered out of 100 who received questionnaires. In the work of Zakeri et al. [25], 185 (61.6%) nurses answered out of 300 who received questionnaires and in the work of Zhang et al. [37], 240 (59.7%) medical workers responded to 402 questionnaires sent out. In the work of Sanso et al. [33], 210 (4.8%) nurses responded to 4336 questionnaires sent out. The response rate to the surveys we distributed (5.5%) was low and comparable to that obtained by Spanish researchers before the COVID-19 pandemic (5%). This might depend on the studied populations.

### 4.1. Implications for Practice

Politicians, directors of medical units and nurse managers should monitor working conditions, strengthen the sense of self-efficacy and professional optimism among nurses, implement appropriate interventions to support nurses and provide methods of solving psychological problems among nurses.

### 4.2. Study Limitations

The data were collected using an online survey and an electronic psychological questionnaire.

The authors of the study had not assessed the level of life satisfaction in Polish nurses before the COVID-19 pandemic, which made it impossible to demonstrate the impact of the COVID-19 pandemic on life satisfaction in the studied population. In the future, there is a need to assess nurses’ life satisfaction.

## 5. Conclusions

The majority of Polish nurses surveyed during the COVID-19 pandemic had a low or medium level of life satisfaction. The low response rate to the survey was most likely due to work overload during the COVID-19 pandemic. Working in a public service profession, a nurse is exposed to stressful factors related to protecting human health and saving lives. Constant difficult and stressful situations, and mental and physical fatigue, often experienced by nursing professionals can contribute to a lack of motivation, occupational burnout, indifference and even mental and physical disease. Further research is necessary to assess the factors positively influencing the level of life satisfaction.

## Figures and Tables

**Table 1:** Characteristics of the nurses.

Socio-Demographic Data of the Participants		N (Total 361)
Gender	Female	342
	Male	19
Age (years)	21–30	57
	31–40	59
	41–50	115
	51–60	119
	≥61	11
Place of residence	City	302
	Village	59
Education	Master of nursing	165
	Bachelor of nursing	103
	Secondary education in nursing	93
Place of work	Outpatient medical clinics	63
	Hospital wards	273
	Hospice, health care and curative institutions	18
	Medical universities	7
Years of employment in the profession (years)	≤5	57
	6–10	32
	11–20	50
	≥21	222

**Table 2:** The nurses’ responses to the statements in the Satisfaction with Life Scale.

Satisfaction with Life	1. In Most Ways, My Life Is Close to My Ideal	2. The Conditions of My Life Are Excellent	3. I Am Satisfied with My Life	4. Thus Far, I have Gotten the Important Things I Want in Life	5. If I could Live My Life over, I would Change almost Nothing
Strongly disagree	23 (6.4%)	14 (3.9%)	7 (1.9%)	12 (3.3%)	37 (10.3%)
Disagree	45 (12.5%)	34 (9.5%)	16 (4.4%)	25 (6.9%)	50 (13.9%)
Slightly disagree	59 (16.3%)	54 (14.9%)	26 (7.2%)	48 (13.3%)	73 (20.2%)
Neither agree nor disagree	135 (37.4%)	107 (29.6%)	60 (16.6%)	59 (16.3%)	68 (18.8%)
Slightly agree	67 (18.6%)	101 (28%)	146 (40.5%)	143 (39.6%)	77 (21.3%)
Agree	26 (7.1%)	40 (11.1%)	77 (21.3%)	53 (14.7%)	38 (10.5%)
Strongly agree	6 (1.7%)	11 (3%)	29 (8.1%)	21 (5.9%)	18 (5%)

**Table 3:** Results of the Satisfaction with Life Scale for nurses.

Level of Satisfaction with Life	Sten Score (Raw Score)	Number of Nurses	Level of Satisfaction with Life in Respondents
Low	1 (5–9)	11 (3.1%)	83 (23%)
	2 (10–11)	7 (1.9%)	
	3 (12–14)	26 (7.2%)	
	4 (15–17)	39 (10.8%)	
Medium	5 (18–20)	74 (20.5%)	159 (44.1%)
	6 (21–23)	85 (23.6%)	
High	7 (24–26)	63 (17.5%)	119 (32.9%)
	8 (27–28)	28 (7.7%)	
	9 (29–30)	14 (3.9%)	
	10 (31–35)	14 (3.8%)	

**Table 4:** Influence of various factors on satisfaction with life.

Socio-Demographic Data		Satisfaction with Life			
		Raw Score	*p*	Sten Score	*p*
Gender	Male	21 (18 ÷ 26)	*p* = 0.981	6 (5 ÷ 7)	*p* = 0.932
	Female	21 (18 ÷ 25)		6 (5 ÷ 7)	
Place of residence	City	21.1 ± 5.7	*p* = 0.618	5.76 ± 1.97	*p* = 0.542
	Village	20.7 ± 5.4		5.59 ± 1.79	
Place of work	Paediatric wards	20 (18 ÷ 22)	*p* = 0.216	5 (5 ÷ 6)	*p* = 0.221
	Internal diseases wards	21 (18 ÷ 26.5)		6 (6 ÷ 7.5)	
	Surgical wards	21 (18 ÷ 25)		6 (5 ÷ 7)	
	Intensive care units	23 (17 ÷ 25)		6 (4 ÷ 7)	
	Emergency departments	22 (19 ÷ 26)		6 (5 ÷ 7)	
	Psychiatric wards for adultsand children	18 (15 ÷ 22)		5 (3.5 ÷ 6)	
Place of work	Outpatient medical clinics	20 (18 ÷ 24)	*p* = 0.137	5 (5 ÷ 7)	*p* = 0.096
	Hospital wards (paediatric wards, surgical wards, internal diseases wards, surgical units, intensive care units for adults, emergency departments, intensive care units for children, psychiatric wards for adults, psychiatric wards for children)	21 (18 ÷ 25)		6 (5 ÷ 7)	
	Hospice, health care and curative institutions	24 (21 ÷ 27)		7 (6 ÷ 8)	
	Medical universities	22 (20 ÷ 23)		6 (5 ÷ 6)	
Years of employment in the profession (years)	≤5	21.5 ± 6.1	*p* = 0.669	5.91 ± 2.15	*p* = 0.513
	6–10	21.8 ± 7.5		6.09 ± 2.48	
	11–20	21.2 ± 5.4		5.78 ± 1.89	
	≥21	20.8 ± 5.3		5.63 ± 1.80	

Data are presented as Mean (X) ± SD, M (25Q ÷ 75Q).

## Data Availability

Data available on request due to privacy restriction.
